# A modelling approach for exploring muscle dynamics during cyclic contractions

**DOI:** 10.1371/journal.pcbi.1006123

**Published:** 2018-04-16

**Authors:** Stephanie A. Ross, Nilima Nigam, James M. Wakeling

**Affiliations:** 1 Department of Biomedical Physiology and Kinesiology, Simon Fraser University, Burnaby, British Columbia, Canada; 2 Department of Mathematics, Simon Fraser University, Burnaby, British Columbia, Canada; Stanford University, UNITED STATES

## Abstract

Hill-type muscle models are widely used within the field of biomechanics to predict and understand muscle behaviour, and are often essential where muscle forces cannot be directly measured. However, these models have limited accuracy, particularly during cyclic contractions at the submaximal levels of activation that typically occur during locomotion. To address this issue, recent studies have incorporated effects into Hill-type models that are oftentimes neglected, such as size-dependent, history-dependent, and activation-dependent effects. However, the contribution of these effects on muscle performance has yet to be evaluated under common contractile conditions that reflect the range of activations, strains, and strain rates that occur *in vivo*. The purpose of this study was to develop a modelling framework to evaluate modifications to Hill-type muscle models when they contract in cyclic loops that are typical of locomotor muscle function. Here we present a modelling framework composed of a damped harmonic oscillator in series with a Hill-type muscle actuator that consists of a contractile element and parallel elastic element. The intrinsic force-length and force-velocity properties are described using Bézier curves where we present a system to relate physiological parameters to the control points for these curves. The muscle-oscillator system can be geometrically scaled while preserving dynamic and kinematic similarity to investigate the muscle size effects while controlling for the dynamics of the harmonic oscillator. The model is driven by time-varying muscle activations that cause the muscle to cyclically contract and drive the dynamics of the harmonic oscillator. Thus, this framework provides a platform to test current and future Hill-type model formulations and explore factors affecting muscle performance in muscles of different sizes under a range of cyclic contractile conditions.

## Author summary

One of the primary functions of skeletal muscle is to generate work and power to move the body during locomotor tasks such as walking and running. Because it is difficult to measure muscle behaviour in living animals, most of what we know about how muscles perform this function is from experiments where the muscle is removed from the animal and studied under controlled laboratory conditions, or from computer simulations of such muscle contractions. Recent work has shown how internal mass within the muscle causes scale-dependent changes to contractile properties. This study demonstrates a forward-dynamic modelling framework that links a Hill-type muscle model to an oscillating external load. Scaling relations are developed to preserve the kinematic and dynamic similarity of the system to allow the model to be implemented from single fibre to whole muscle sizes. The model replicates contraction cycles that are typically seen in real muscles. The framework will allow the relative effects of history-dependent, internal mass and activation properties to be quantitatively evaluated for cyclic contractions.

This is a *PLOS Computational Biology* Methods paper.

## Introduction

One of the primary functions of skeletal muscle is to perform work by cyclically contracting to move an external load during locomotion. For the last half-century, experimental work-loop studies have provided insight into how muscle force and length, and thus work, depend on interactions between neural excitation and the external load placed on the muscle during cyclic contractions. These interaction effects are supported by early *in vitro* studies examining the behaviour of invertebrate flight muscles coupled to external loads with different elastic, viscous and inertial properties [1–2]. More recently, *in vivo* studies on birds such as turkeys [3] and guinea fowl [4], and larger vertebrates such as wallabies [5] and goats [6], have shown that altering the characteristics of the external environment can substantially change the work a muscle can do per contraction cycle. For example, [3] found that muscle fascicles within the lateral gastrocnemius muscle in turkeys behave like a motor during uphill running by generating large forces for the duration of the shortening phase of the cycle, and act like a strut during level running by minimizing their shortening while the force is high. Thus, the behaviour of muscle depends on the demands of the task in addition to the properties of the muscle.

While work-loop studies have provided insight into how changes in neural excitation and external conditions alter the behaviour of muscle during cyclic contractions, the contribution of the mechanical properties of the muscle itself remain largely unknown. Much of what we know about the mechanisms that underlie muscle contractile behaviour is from measures on small muscles or single fibres during maximal contractions under constant load. Furthermore, the Hill-type muscle models that are used to predict and understand muscle behaviour rely on the assumption that these mechanisms seen in small muscles or fibres under controlled conditions are the same as that in large whole muscles during submaximal cyclic contractions under varying load. However, these models have limited accuracy, particularly during cyclic contractions at the submaximal levels of activation that typically occur during locomotion and other daily activities [7–11]. Most recently, Dick and colleagues [11] tested Hill-type model predictions of human gastrocnemius forces during cycling against measured ultrasound and electromyography data and found that while model errors were low for slow contractions at high activations, errors became substantially larger with increasing contraction speed and decreasing activation. Thus, Hill-type models are currently unable to consistently replicate the salient features of muscle contractile performance in humans and animals.

To improve the ability of Hill-type models to mimic whole muscle behaviour *in vivo*, recent studies have incorporated effects into these models that are typically neglected. Wakeling and colleagues [12] developed a Hill-type model that allowed for independent recruitment of fast and slow contractile elements. When compared to models with single contractile elements or models with fast and slow elements that followed an orderly recruitment pattern, the differential recruitment model was most accurate in predicting *in situ* [12] and *in vivo* [10] goat muscle forces. In addition to fibre recruitment, a muscle’s force depends on its previous length and rate of length change [13–17]. When incorporated into the Hill-type muscle actuators of multibody musculoskeletal models, these history-dependent effects have been shown to substantially alter muscle power predictions in simulations of cycling [18], and muscle force magnitude and timing predictions in simulations of countermovement jumping [19]. Other such overlooked effects include inertia due to muscle tissue mass and tendon or serial elastic element (SEE) dynamics. Ross and Wakeling [20] found that adding mass to a Hill-type model leads to slower maximum contraction speeds, and this effect is more pronounced for larger muscles and lower levels of activation, and Curtin and others [21] found that including a compliant SEE in the model formulation improves muscle force predictions during sinusoidal contractions. Further modelling studies have shown that serial compliance amplifies the maximum power a muscle can deliver to an inertial load [22], and varying the magnitude of this compliance substantially alters estimates of muscle efficiency during locomotor tasks such as walking and running [23]. Together these findings show promise for improving our understanding of muscle function and our ability to use Hill-type models to predict *in vivo* muscle forces.

Despite the potential for improving Hill-type models by incorporating these different effects, their influence on muscle performance has yet to be evaluated under common contractile conditions that reflect the range of activations, strains, and strain rates that occur in real muscle. Herein we present a novel forward dynamics framework that consists of a Hill-type muscle actuator in series with a damped harmonic oscillator to represent the physical load placed on the muscle during locomotion. The system is driven by time-varying activation of the muscle actuator to simulate the contraction cycles seen *in vivo*.

## Methods and models

**Fig 1:**
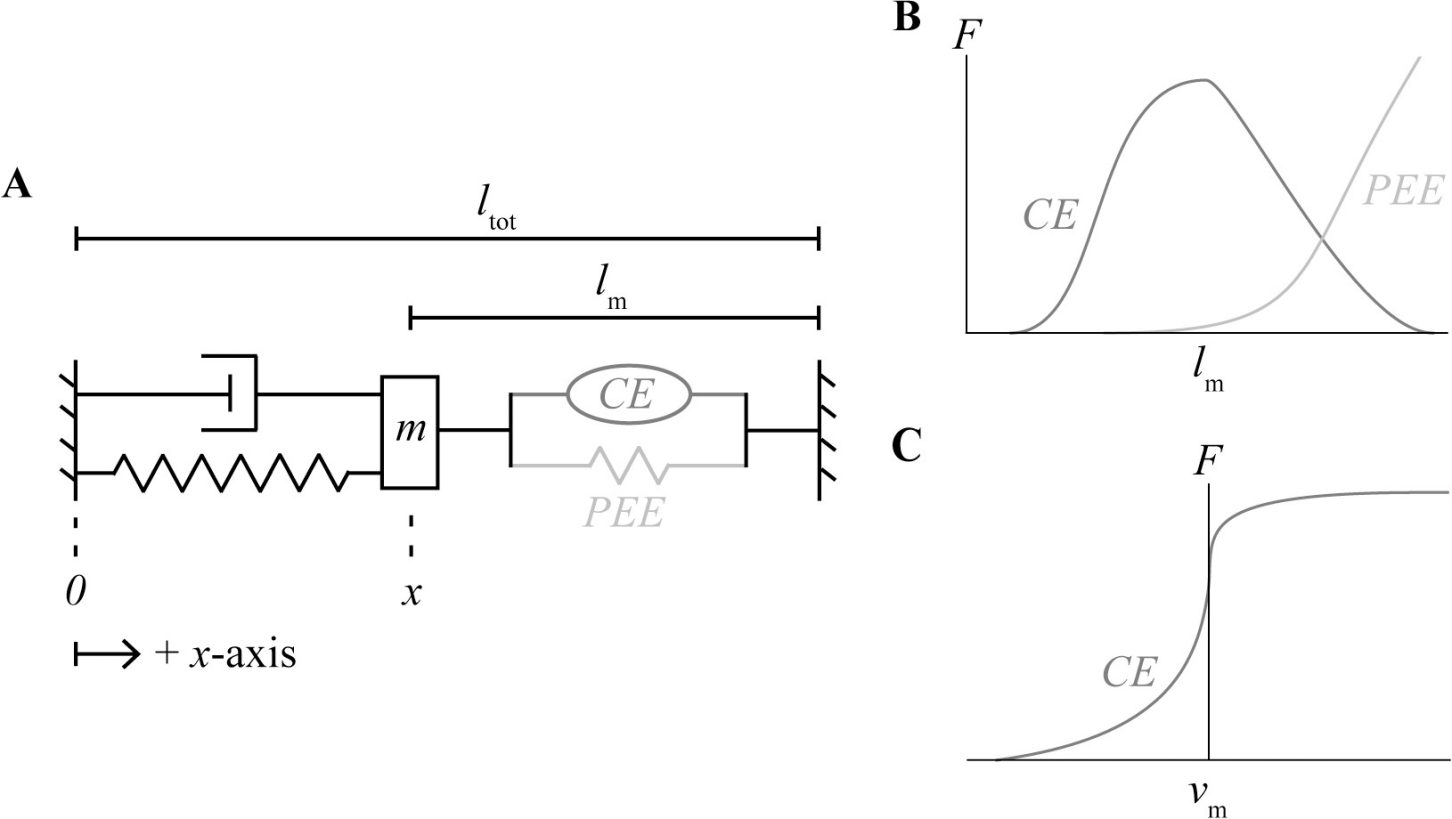
Visualization of modelling framework. Second-order dynamic system composed of a Hill-type muscle model in series with a damped harmonic oscillator (A). The force of the muscle is given by the sum of the active force due to the contractile element (CE) as a function of its length (B) and velocity (C), and the passive force due to the parallel elastic element (PEE) as a function of its length (B).

The model system is composed of a Hill-type muscle model in series with a damped harmonic oscillator (Fig 1). The Hill-type model contains a contractile element and a parallel elastic element, and does not account for the effects of a tendon. The muscle is assumed to only contain parallel fibres that generate force along the longitudinal *x*-axis of the system. The length of the muscle *l*_m_ is equal to the sum of the fixed total length of the system *l*_tot_ and the position *x* of the oscillator mass *m*:

$$l_{m}=l_{tot}-x$$ (1)

The motion of the system is constrained to be one-dimensional along the longitudinal axis of the muscle. The dynamics of the system can be described by:

$$\Sigma F=F_{m}-F_{s}-F_{d}$$ (2)

where ∑*F* is the sum of the forces acting on the mass, *F*_m_ is the muscle force, *F*_s_ is the spring force, and *F*_d_ is the damping force. *F*_s_ is linearly dependent on the displacement Δ*x* of the mass, *m*:

$$F_{s}=k\Delta x$$ (3)

where *k* is the stiffness coefficient and Δ*x* is equal to the difference between *x* and the resting length of the oscillator *x*_0_. The force of the viscous damper is given by:

$$F_{d}=b\frac{d\Delta x}{dt}$$ (4)

where *b* is the damping coefficient. *F*_m_ is given by:

$$F_{m}={aF}_{A}F_{V}+F_{P}$$ (5)

where *F*_A_ and *F*_P_ are the active and passive forces as a function of the dimensionless muscle length ${\hat{l}}_{m}$, respectively, and *F*_V_ is the active force as a function of the dimensionless muscle velocity ${\hat{v}}_{m}$. ${\hat{l}}_{m}$ is calculated as *l*_m_ normalized by the muscle optimal length *l*_0_:

$${\hat{l}}_{m}=\frac{l_{m}}{l_{0}}$$ (6)

and ${\hat{v}}_{m}$ is calculated as the first time derivative of ${\hat{l}}_{m}$ normalized by the maximum unloaded shortening strain rate, ${\dot{\varepsilon }}_{0}$:

$${\hat{v}}_{m}=\frac{\frac{d}{dt}(\frac{l_{m}}{l_{0}})}{{\dot{\varepsilon }}_{0}}$$ (7)

By convention, *l*_0_ is defined as the muscle length corresponding to the maximum isometric force and ${\dot{\varepsilon }}_{0}$ is the maximum shortening strain rate and is equal to the maximum of the first derivative of ${\hat{v}}_{m}$ with respect to time. Combining and rearranging Eqs (2–5) gives:

$$m\frac{d^{2}\Delta x}{dt^{2}}+b\frac{d\Delta x}{dt}+k\Delta x={aF}_{A}F_{V}+F_{P}$$ (8)

The model is driven with time-varying excitation *u* that determines the activation *a* by solving the following first-order bilinear differential equation [24]:

$$\frac{da}{dt}+a\;[\frac{1}{\tau_{act}}\left(\beta +u(1-\beta )\right)]=\frac{1}{\tau_{act}}u$$ (9)

where τ_act_ is the activation time constant to account for the delay between onset of excitation and maximum twitch force, *β* is the ratio between τ_act_ and the deactivation time delay, and *u* is muscle excitation. *u* is represented by a repeating square wave function with a characteristic frequency, *f*, and duty cycle, *D*.

### Root model properties

The root muscle properties were chosen to represent a bundle of parallel muscle fibres that generate force strictly along the length of the model. The maximum isometric force, *F*_0_, of the root model is calculated as the product of the maximum isometric stress, *σ*_0_, and the muscle cross-sectional area, *A*:

$$F_{0}=\sigma_{0}A$$ (10)

The model is assumed to be cylindrical in shape so *A* is given by:

$$A=\frac{\pi }{4}{\left(\frac{l_{0}}{R}\right)}^{2}$$ (11)

where *R* is the aspect ratio between *l*_0_ and the diameter of the muscle model. The mass of the muscle *m*_m_ is the product of the muscle density *ρ* and the volume, and the volume is the product of *l*_0_ and *A*. Therefore:

$$m_{m}=\rho l_{0}A$$ (12)

While the properties of the muscle can be taken from empirical data, determining the properties of the harmonic oscillator is less straightforward because the external loads applied to muscle *in vivo* cannot be resolved into their individual components such as limb inertia, passive elasticity, and gravitational forces. Therefore, the properties of the harmonic oscillator are chosen so that the kinematics and dynamics of the muscle model replicate the behaviour of muscle *in vivo*.

Given that one of the primary functions of muscle is to generate power, we chose the base properties of the harmonic oscillator that maximize the average mass-specific mechanical power output of the muscle per cycle P* [25]. Power is the rate of doing work, and the net work of the muscle is given by the integral of the muscle force over the length change, so P^*^ can be calculated as the product of the net mechanical muscle work output per cycle and the frequency *f*, divided by the muscle mass *m*_m_:

$$P^{*}=\frac{\int F_{m}(dl_{m})f}{m_{m}}$$ (13)

Consult [26] for further explanation of P^*^. Because the muscle and harmonic oscillator are mechanically coupled in our model, both *F*_m_ and the change in *l*_m_ per cycle, and therefore P^*^, depend on the chosen values of oscillator mass *m*, damping coefficient *b*, and spring stiffness coefficient *k*. Although these parameters can be solved for directly using optimization, we have chosen instead to link them to the properties of the muscle to reduce the number of unknown parameters and provide values with greater physiological meaning. Because the muscle and harmonic oscillator are connected in series, the change in *l*_m_ per cycle will be maximal when the amplitude of the oscillator displacement is maximal. For a simple harmonic oscillator without damping, this maximal amplitude occurs when the natural frequency *ω*_n_ is equal to the driving frequency *ω*_d_, where *ω*_n_ is given by:

$$\omega_{n}=\sqrt{\frac{k}{m}}$$ (14)

However, for a driven oscillator with damping, the maximal amplitude occurs when *ω*_n_ is a fraction *c*_1_ of *ω*_d_ [27]:

$$\omega_{n}=c_{1}\omega_{d}$$ (15)

Converting *ω*_d_ from an angular to a temporal frequency *f*_d_ gives:

$$\omega_{n}=2\pi c_{1}f_{d}$$ (16)

Combining Eq (14) and Eq (16) gives an expression for *k* in terms of *c*_1_, *m*, and *f*_d_:

$$k=m{\left(2\pi c_{1}f_{d}\right)}^{2}$$ (17)

To relate *b* to *c*_1_, *m*, and *f*_d_ we can constrain *b* to be at a critical level (for a critically damped system) such that:

$$b=\sqrt{4mk}$$ (18)

Substituting Eq (17) into Eq (18) gives:

$$b=4\pi mc_{1}f_{d}$$ (19)

To relate the inertial load due to mass *m* to the maximum isometric force *F*_0_, we can set *m* to be equal to a fraction *c*_2_ of *F*_0_:

$$m=c_{2}F_{0}$$ (20)

Substituting Eq (20) into Eq (17) and Eq (19) gives:

$$k=c_{2}F_{0}{\left(2\pi c_{1}f_{d}\right)}^{2}$$ (21)

and

$$b=4\pi c_{2}F_{0}c_{1}f_{d}$$ (22)

In addition to the spring stiffness *k*, the force applied to the muscle due to the spring also depends on the resting length *x*_0_ of the harmonic oscillator (Eq 3). If at rest *l*_m_ is equal to *l*_0_, and the total length of the system *l*_tot_ is the sum of the lengths of the oscillator and the muscle (Eq 1), then the oscillator length when the muscle is at *l*_0_ is equal to *l*_tot_ minus *l*_0_. If we set *x*_0_ to be a fraction *c*_3_ of the oscillator length when *l*_m_ is *l*_0_, then:

$$x_{0}=c_{3}(l_{tot}-l_{0})$$ (23)

High P^*^ would occur for contractions that have a high activation throughout shortening, but minimal activation during lengthening of the muscle. Thus, the value of P^*^ also depends on the duty cycle *D* that represents the fraction of each excitation cycle where the muscle is excited [28], as well as the activation dynamics that govern how rapidly the activation state can be turned on and off for the shortening phase. The unknown parameters *c*_1_, *c*_2_, *c*_3_, *f*_d_ and *D* were optimized for by maximizing the model output P^*^ using nonlinear global optimization for a fast muscle with ${\dot{\varepsilon }}_{0}$ of 10 s^-1^ and *u*_max_ of 1. Values of the model and equation parameters can be found in Table 1.

**Table 1:** Model and equation parameters.

Parameter	Definition	Value	Source
*l*_0_	Muscle optimal length	0.02 m	Estimated from literature [29]
*σ*_0_	Muscle maximum isometric stress	225000 Pa	Estimated from literature [30]
*ρ*	Muscle density	1060 kg m^-3^	Literature [31]
*R*	Muscle aspect ratio (*l*_0_:diameter)	100	
*A*	Muscle cross-sectional area	Varied	Calculated (Eq 11)
*F*_0_	Muscle maximum isometric force	Varied	Calculated (Eq 10)
*m*_m_	Muscle mass	Varied	Calculated (Eq 12)
τ_act_	Time constant for activation	0.045 s for ${\dot{\varepsilon }}_{0}$ of 5 s^-1^; 0.025 s for ${\dot{\varepsilon }}_{0}$ of 10 s^-1^	Literature [11]
*β*	Ratio of τ_act_ to deactivation time constant	0.6	Literature [11]
*D*	Excitation duty cycle	S1 Table	Optimized
*f*_d_	Driving frequency	S1 Table	Optimized
*c*_1_	Ratio between *ω*_n_ and *ω*_d_	S1 Table	Optimized
*c*_2_	Ratio between *m* and *F*_0_	S1 Table	Optimized
*c*_3_	Ratio between *x*_0_ and *l*_0_	S1 Table	Optimized
*m*	Oscillator mass	Varied	Calculated (Eq 20)
*k*	Oscillator stiffness coefficient	Varied	Calculated (Eq 21)
*b*	Oscillator damping coefficient	Varied	Calculated (Eq 22)
*x*_0_	Resting length of oscillator	Varied	Calculated (Eq 23)
*l*_tot_	Total length of model	2 *l*_0_	

### Model non-dimensionalization and scaling

The standard Hill-type formulation assumes that whole muscles behave as single fibres, with the muscle forces scaling with cross-sectional area and muscle lengths scaling with optimal length. However, it has been shown that the presence of mass in muscle causes a scale-dependent distortion that limits this assumption [20]. To explore the contribution of these scale-dependent distortions in muscles of different sizes, the model framework must be able to scale geometrically while preserving kinematic and dynamic similarity. In other words, a larger muscle would have greater forces, lengths and power outputs, but the non-dimensional forms of these parameters should remain the same. To achieve this, the spring-mass-damper properties of the damped harmonic oscillator must be adjusted to preserve kinematic and dynamic similarity of the whole system. For kinematic and dynamic similarity to occur, all dimensionless parameter groups, shown in curly brackets, are held constant for simulations with different geometric scales. To identify these dimensionless parameter groups, the dimensional system variables must be non-dimensionalized using methods presented in [32].

Eq (9) contains 3 dimensional variables to be normalized: *a*, *u*, and *t*. Because *u* is defined as a square wave that varies in amplitude between 0 and 1, *u* and *a* in Eq (9) can be denoted as $\hat{u}$ and $\hat{a}$, respectively. The remaining time variable *t* can be normalized by a muscle time scale, *t*_m_:

$$\hat{t}=\frac{t}{t_{m}}$$ (24)

This gives the following equation:

$$\frac{d\hat{a}}{d\hat{t}}+\{\frac{t_{m}∙\beta }{\tau_{act}}\}\hat{a}\left(1-\hat{u}\right)=\{\frac{t_{m}}{\tau_{act}}\}\hat{u}\left(1-\hat{a}\right)$$ (25)

Since the excitation input is prescribed as a normalized value, $\hat{u}$ can be scaled using:

$$\hat{u}=\frac{u}{u_{max}}$$ (26)

where *u*_max_ is the maximum possible muscle excitation for a given simulation.

Eq (8) contains 5 dimensional variables: *F*_A_, *F*_P_, *F*_V_, Δ*x*, and *t*. The forces *F*_A_, *F*_P_ and *F*_V_ are normalized by the maximum isometric force, *F*_0_:

$${\hat{F}}_{A}=\frac{F_{A}}{F_{0}}$$ (27)

$${\hat{F}}_{P}=\frac{F_{P}}{F_{0}}$$ (28)

$${\hat{F}}_{V}=\frac{F_{V}}{F_{0}}$$ (29)

The displacement of the harmonic oscillator, Δ*x*, is normalized with its resting length, *x*_0_:

$$\Delta \hat{x}=\frac{\Delta x}{x_{0}}$$ (30)

and *t* is normalized with an oscillator time scale, *t*_h_:

$$\hat{t}=\frac{t}{t_{h}}$$ (31)

Both *t*_h_ and *t*_m_ are set to a value of 1 as there is no experimental evidence to suggest that muscle behaviour scales in the time dimension. Combining Eq (9) and Eqs (26–31) gives the equation of motion:

$$\{\frac{mx_{0}}{{{F_{0}t}_{h}}^{2}}\}[\frac{d^{2}\Delta \hat{x}}{d{\hat{t}}^{2}}+\{\frac{bt_{h}}{m}\}\frac{d\Delta \hat{x}}{d\hat{t}}+\{\frac{k{t_{h}}^{2}}{m}\}\Delta \hat{x}]=F_{0}u_{max}\left[{\hat{a}\hat{F}}_{A}{\hat{F}}_{V}+{\hat{F}}_{P}\right]$$ (32)

To ensure dynamic similarity between the root and scaled model, the dimensionless groups (curly brackets) from Eq (32) must remain constant:

$$\frac{m_{r}∙x_{0,r}}{F_{0,r}{{∙t}_{h,r}}^{2}}=\frac{m_{s}∙x_{0,s}}{F_{0,s}{{∙t}_{h,s}}^{2}}$$ (33)

$$\frac{b_{r}∙t_{h,r}}{m_{r}}=\frac{b_{s}∙t_{h,s}}{m_{s}}$$ (34)

$$\frac{k_{r}∙{t_{h,r}}^{2}}{m_{r}}=\frac{k_{s}∙{t_{h,s}}^{2}}{m_{s}}$$ (35)

where the subscripts *r* and *s* indicate the root and scaled models, respectively.

We can define the following scaling factors for each parameter, *p*, as the ratio *λ* between the parameter value of the scale model and root model:

$$\lambda_{p}=\frac{p_{s}}{p_{r}}$$ (36)

Rearranging Eqs (33–35) and substituting in the scaling factors gives the following scaling laws:

$$\lambda_{m}∙\lambda_{x_{0}}=\lambda_{t_{h}}∙\lambda_{F_{0}}$$ (37)

$$\lambda_{b}∙\lambda_{t_{h}}=\lambda_{m}$$ (38)

$$\lambda_{k}∙{\lambda_{t_{h}}}^{2}=\lambda_{m}$$ (39)

To ensure geometric similarity between models, the muscle length scale factor is set equal to the harmonic oscillator length scale:

$$\lambda_{x_{0}}=\lambda_{l_{0}}$$ (40)

To ensure kinematic similarity, the velocity of the muscle must scale with the velocity of the harmonic oscillator. The muscle velocity scale factor is proportional to the muscle length scale factor $\lambda_{l}{}_{{}_{0}}$ divided by the muscle time scale factor $\lambda_{t}{}_{{}_{m}}$, and the oscillator velocity scale factor is equal to the oscillator length scale factor $\lambda_{x}{}_{{}_{0}}$ divided by the oscillator time scale factor $\lambda_{t}{}_{{}_{h}}$. This leads to:

$$\frac{\lambda_{x_{0}}}{\lambda_{t_{h}}}=\frac{\lambda_{l_{0}}}{\lambda_{t_{m}}}$$ (41)

Combining and rearranging Eqs (40) and (41) gives:

$$\lambda_{t_{h}}=\lambda_{t_{m}}$$ (42)

To solve for the remaining scale factors in terms of $\lambda_{l}{}_{{}_{0}}$, additional assumptions must be introduced. Experimental evidence suggests that the maximum isometric stress of skeletal muscle is roughly constant across a range of animals, and does not appear to scale with muscle or animal size [30]. Therefore, we can assume that the maximum isometric stress *σ*_0_ is constant and $\lambda_{\sigma }{}_{{}_{0}}$ is equal to 1. Stress is calculated as force over cross-sectional area which gives:

$$\lambda_{\sigma_{0}}=\frac{\lambda_{F_{0}}}{{\lambda_{l_{0}}}^{2}}$$ (43)

Substituting in the value of $\lambda_{\sigma }{}_{{}_{0}}$ leads to:

$$\lambda_{F_{0}}={\lambda_{l_{0}}}^{2}$$ (44)

In addition to stress, muscle density *ρ* is typically assumed to remain constant across muscles of different sizes [25], and therefore the muscle density scale factor *λ*_*ρ*_ is equal to 1. Density is calculated as mass divided by volume, and since the model is geometrically scaled, the change in volume is proportional to the change in length cubed:

$$\lambda_{\rho }=\frac{\lambda_{m_{m}}}{{\lambda_{l_{0}}}^{3}}$$ (45)

Solving for the muscle mass scale factor $\lambda_{m}{}_{{}_{m}}$ gives:

$$\lambda_{m_{m}}={\lambda_{l_{0}}}^{3}$$ (46)

The average mass-specific power output per cycle P^*^ at a given cycle frequency is also relatively constant across a range of vertebrate muscles [25]. Therefore:

$$\lambda_{F_{0}}\lambda_{l_{0}}=\lambda_{t_{m}}\lambda_{m_{m}}$$ (47)

Combining Eqs (44), (46) and (47) gives:

$$\lambda_{t_{m}}=1$$ (48)

and combining Eqs (42) and (48) gives:

$$\lambda_{t_{h}}=1$$ (49)

Substituting Eqs (40), (44) and (49) into Eq (37) results in an expression for *λ*_*m*_ in terms of $\lambda_{l}{}_{{}_{0}}$:

$$\lambda_{m}=\lambda_{l_{0}}$$ (50)

Similarly, an expression for *λ*_*b*_ in terms of $\lambda_{l}{}_{{}_{0}}$ can be found by substituting Eqs (49) and (50) into Eq (38):

$$\lambda_{b}=\lambda_{l_{0}}$$ (51)

Combining Eqs (39), (49) and (50) gives:

$$\lambda_{k}=\lambda_{l_{0}}$$ (52)

A summary of the scaling factors values can be found in Table 2.

**Table 2:** Model scaling factors.

Scaling factor	Value	Source
$\lambda_{l}{}_{{}_{0}}$	1 or 10	Varied
$\lambda_{\sigma }{}_{{}_{0}}$	1	Literature [30]
*λ*_*ρ*_	1	Literature [25]
*λ*_P*_	1	Literature [25]
$\lambda_{x}{}_{{}_{0}}$	$\lambda_{l}{}_{{}_{0}}$	Calculated (Eq 40)
$\lambda_{F}{}_{{}_{0}}$	$\lambda_{l}{{}_{{}_{0}}}^{2}$	Calculated (Eq 44)
$\lambda_{m}{}_{{}_{m}}$	$\lambda_{l}{{}_{{}_{0}}}^{3}$	Calculated (Eq 46)
$\lambda_{t}{}_{{}_{m}}$	1	Calculated (Eq 48)
$\lambda_{t}{}_{{}_{h}}$	1	Calculated (Eq 49)
*λ*_*m*_	$\lambda_{l}{}_{{}_{0}}$	Calculated (Eq 50)
*λ*_*b*_	$\lambda_{l}{}_{{}_{0}}$	Calculated (Eq 51)
*λ*_*k*_	$\lambda_{l}{}_{{}_{0}}$	Calculated (Eq 52)

### Force-velocity and force-length curves

A variety of different functions have been used to represent the intrinsic force-velocity and force-length relationships, including piecewise [33–36], polynomial [35–38], hyperbolic [39–40], trigonometric [41–42], logarithmic [40], and exponential [34,39,43] functions. There is typically a trade-off between accuracy and cost when choosing curves to model these intrinsic properties. For example, piecewise functions typically provide the best physiological representation but they can create computational issues due to singularities in the first derivative of the function within the operating range of muscle lengths and velocities. In contrast, polynomials are smooth continuous functions that are easy to implement, however, they are typically poor at representing the behaviour of muscle outside of the usual operating range. This particularly becomes an issue for forward dynamics simulations where the lengths and velocities can be unconstrained.

**Fig 2:**
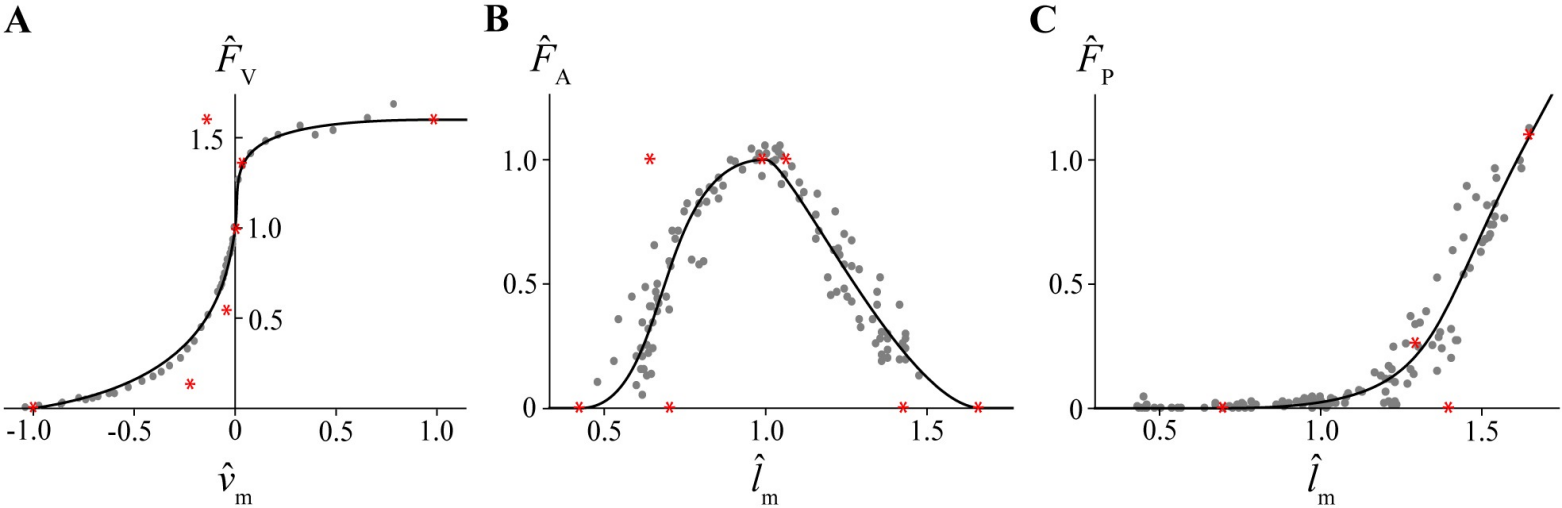
Force-velocity and force-length curves. Normalized force-velocity (A), active force-length (B) and passive force-length (C) curves (black lines). The force-velocity curve and the active and passive force-length curves are fitted to experimental data from [47] and [48], respectively (grey points). The Bézier control points for each curve are shown as red asterisks.

Bézier splines have been presented as an alternative formulation that provides both improved accuracy and computational efficiency over traditional representations of force-velocity and force-length curves [9]. These functions are parametric curves based on a set of polynomials that smoothly interpolate user-defined control points. For further details on the characteristics and formulation of Bézier curves, consult [44] and [45]. For this study, we used composite cubic Bézier curves to represent the force-velocity and active force-length relationships, and a single cubic Bézier curve to represent the passive force-length relationship (Fig 2). We chose to use composite cubic curves rather than quintic curves as in [9] as they allow more local control when relating the control points for the Bézier curve description to the physiological constraints within empirical muscle data.

The normalized force-velocity curve (Fig 2A) is composed of two cubic Bézier curves joined at a normalized muscle velocity ${\hat{v}}_{m}$ of 0 and normalized muscle force ${\hat{F}}_{m}$ equal to the maximum isometric force *F*_0_. The concentric portion of the curve intersects with the $‑{\hat{v}}_{m}$ axis at the maximum shortening strain rate ${\dot{\varepsilon }}_{0}$, corresponding to ${\hat{v}}_{m}$ of -1, and is symmetric about the line ${\hat{F}}_{m}=‑{\hat{v}}_{m}$ consistent with Hill’s hyperbolic force-velocity curve [46]. The eccentric portion of the curve passes through and plateaus at a ${\hat{F}}_{m}$ value equal to the maximum eccentric force *F*_max_. The normalized force-velocity curve is linearly extrapolated for the extreme ${\hat{v}}_{m}$ values less than -1 and greater than 1, where ${\hat{F}}_{m}$ is set to be equal to 0 and *F*_max_, respectively. To achieve continuity of the curve’s first derivative, the slope of the eccentric and concentric portions of the curve are constrained to be equal where they meet at isometric ${\hat{v}}_{m}$. Additionally, the slope of the curve at a ${\hat{v}}_{m}$ value of 1 and ${\hat{F}}_{m}$ of *F*_max_ is constrained to be equal to zero. Given these experimentally-derived physiological constraints, it is not possible to maintain C1-continuity of the concentric portion of the curve at ${\dot{\varepsilon }}_{0}$; however, the presented curve is C1-continuous throughout the physiological range of ${\hat{v}}_{m}$.

The normalized active force-length curve (Fig 2B) is composed of two cubic Bézier curves representing the ascending and descending limbs joined at optimal length and maximum isometric force which corresponds to a normalized muscle length ${\hat{l}}_{m}$ and ${\hat{F}}_{m}$ of 1. The slopes of both the ascending and descending limbs at *l*_0_ are constrained to be equal to 0, so the first derivative of the curve is continuous at this point. Similarly, the slope of the curve is set to 0 at the start of the ascending limb and at the end of the descending limb so that the curve is C1-continous and the end points beyond where the value of ${\hat{F}}_{m}$ is set to zero.

In contrast to the force-velocity and active force-length curves, the normalized passive force-length curve (Fig 2C) is a single cubic Bézier curve. ${\hat{F}}_{m}$ is set to 0 for ${\hat{l}}_{m}$ less than or equal to ${\hat{l}}_{m}$ of 0.7. The curve is also linearly extrapolated for lengths longer than ${\hat{l}}_{m}$ of 1.65, with the slope of the extrapolated region being equal to the slope of the line between the last and second to last control points. Matching the slopes on either side of the first and last control points guarantees continuity of the passive force-length curve and its first derivative.

The unconstrained degrees of freedom of the force-velocity and force-length relationships were determined by fitting the curves to experimental data from [47] and [48], respectively, by minimizing the coefficient of determination r^2^ using numerical nonlinear global optimization.

### Numerical simulations

To provide a computational proof of our methods, we tested the model at different excitation frequencies *f*, maximum excitation *u*_max_, maximum shortening strain rates ${\dot{\varepsilon }}_{0}$, and muscle length scale factors $\lambda_{l}{}_{{}_{0}}$. The value of *u*_max_ was either 0.1 or 1 to simulate a muscle contracting at 10% and 100% of maximal excitation, respectively. The contractile element of the model behaved as either an entirely fast muscle with a ${\dot{\varepsilon }}_{0}$ of 10 s^-1^ or an entirely slow muscle with a ${\dot{\varepsilon }}_{0}$ of 5 s^-1^. $\lambda_{l}{}_{{}_{0}}$ was either 1 or 10, where the models with $\lambda_{l}{}_{{}_{0}}$ of 1 had the geometric dimensions of the root model. Finally, *f* was set to a value of either 0.5, 1 or 2 Hz.

A single set of forward dynamic simulations were run for each possible combination of *f*, *u*_max_, ${\dot{\varepsilon }}_{0}$ and $\lambda_{l}{}_{{}_{0}}$. The output muscle force *F*_m_, velocity *v*_m_, and length *l*_m_ were measured from the steady-state solution of the system. Due to the presence of damping, the steady-state solution does not depend on initial conditions, unlike the transient solution. The instantaneous muscle power was calculated as the product of *F*_m_ and *v*_m_, and the average mass-specific power per cycle P^*^ was calculated as in Eq (38). All simulations were performed in Wolfram Mathematica Version 11.1.1 [49].

## Results and discussion

In this study, we presented a novel forward dynamics framework that consists of a damped harmonic oscillator in series with a Hill-type muscle actuator driven by time-varying activation. We also provided a description of how to build and implement Bézier splines to represent the intrinsic force-length and force-velocity properties of muscle. The r^2^ for each fitted curve was greater than 0.87, comparable to the r^2^ values for curves from [34,37–42] fitted to the same experimental data from [47–48]. However, the Bézier splines improve upon these more commonly used curves by allowing greater control in replicating the physiological features found in experimental muscle data.

**Fig 3:**
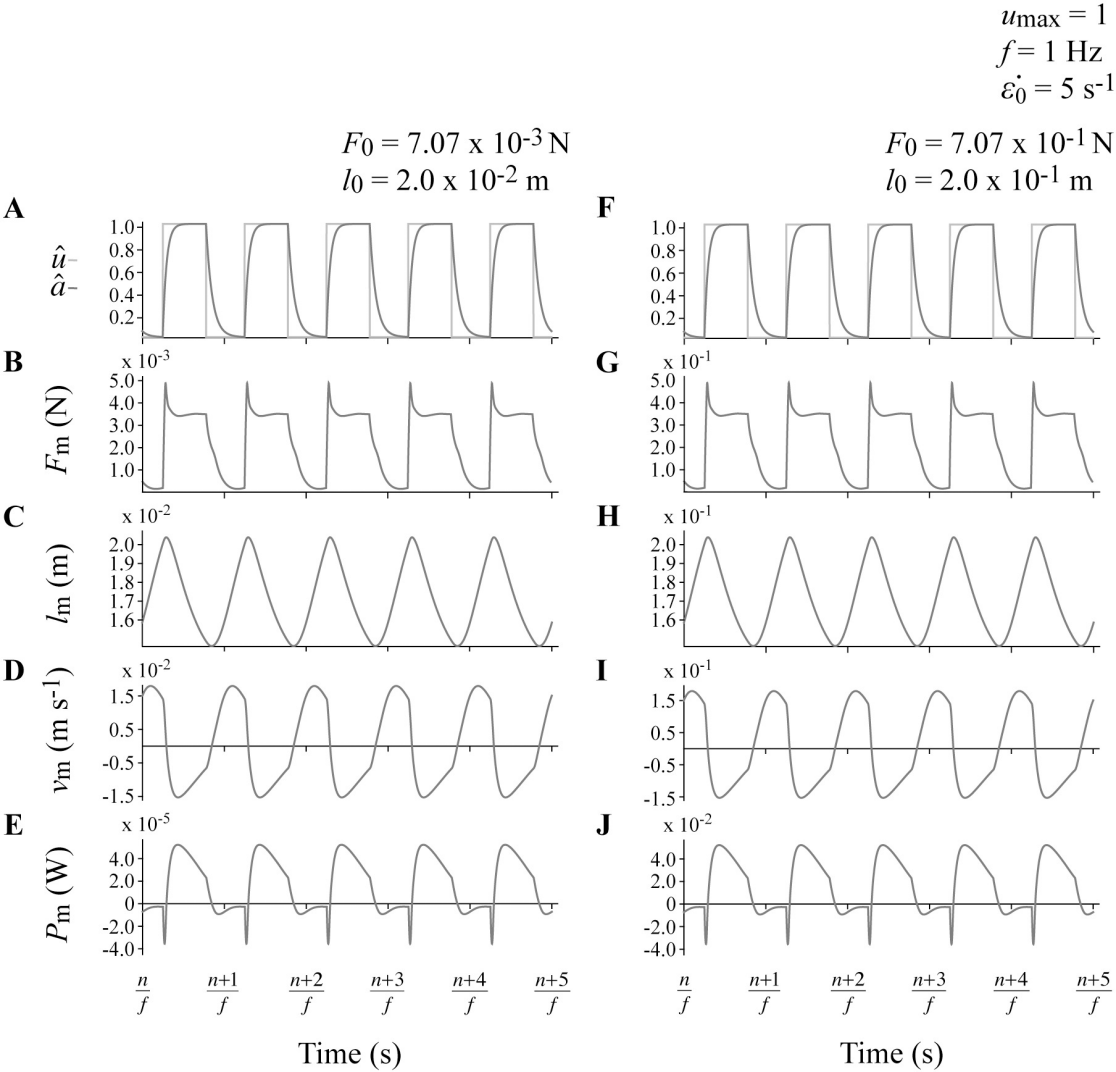
Sample raw output traces. Muscle excitation and activation (A,F), force (B,G), length (C,H), velocity (D,I) and power (E,J) traces for two representative simulations with *u*_max_ of 1, *f* of 1 Hz, ${\dot{\varepsilon }}_{0}$ of 5 s^-1^, and $\lambda_{l}{}_{{}_{0}}$ of 1 (A-E) and 10 (F-J). *n* denotes the cycle number.

We additionally provided methods to geometrically scale the system while preserving kinematic and dynamic similarity. Increasing $\lambda_{l}{}_{{}_{0}}$ from 1 to 10 altered the dimensional dynamics and kinematics of the model, with muscle lengths *l*_m_ and velocities *v*_m_ scaling with $\lambda_{l}{}_{{}_{0}}$, muscle forces *F*_m_ scaling with $\lambda_{l}{{}_{{}_{0}}}^{2}$, and the muscle powers scaling with $\lambda_{l}{{}_{{}_{0}}}^{3}$ (Fig 3). However, the dimensionless output variables for different values of $\lambda_{l}{}_{{}_{0}}$ were identical for simulations with the same *f*, *u*_max_ and ${\dot{\varepsilon }}_{0}$ values, including P^*^ (Table 3). Geometrically scaling the system allows this framework to be used in future to investigate the effects of muscle size while controlling for the dynamic behaviour of the damped harmonic oscillator. Correctly modeling muscle size is important when scaling muscle data from single fibres to predict the function of whole muscles in animals and humans, and is even more important for predicting the function of large extinct species such as dinosaurs.

**Table 3:** Output mass-specific mechanical power output P^*^ for all simulations.

*f* (Hz)	${\dot{\varepsilon }}_{0}$ (s^-1^)	*u*_max_	$\lambda_{l}{}_{{}_{0}}$	P^*^ (W kg^-1^)
0.5	5	0.1	1	1.22
0.5	5	0.1	10	1.22
0.5	5	1	1	20.18
0.5	5	1	10	20.18
1	5	0.1	1	1.43
1	5	0.1	10	1.43
1	5	1	1	25.55
1	5	1	10	25.55
1	10	0.1	1	2.03
1	10	0.1	10	2.03
1	10	1	1	40.60
1	10	1	10	40.60
2	10	0.1	1	1.14
2	10	0.1	10	1.14
2	10	1	1	25.85
2	10	1	10	25.85

**Fig 4:**
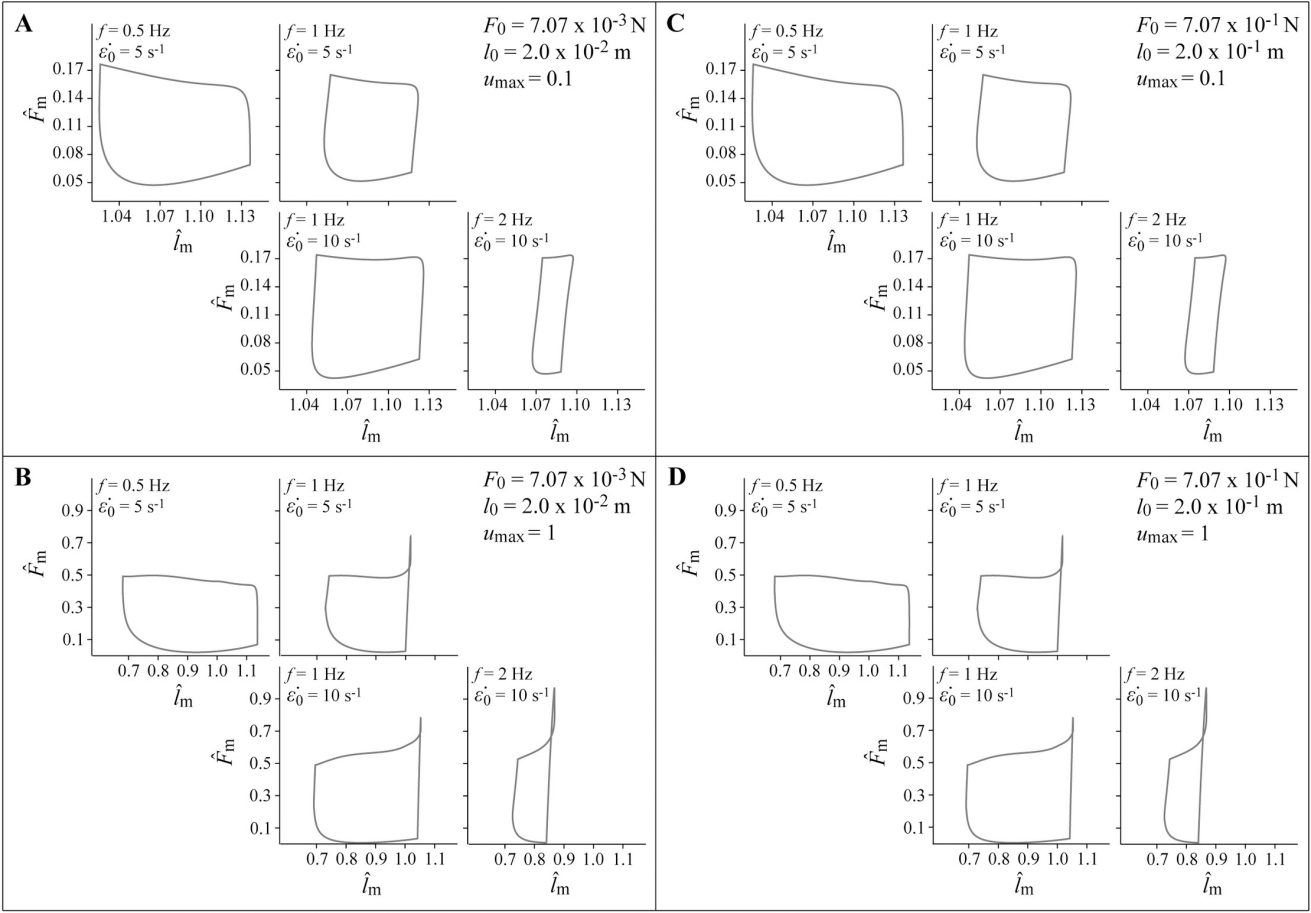
Output simulation work-loops. Muscle work-loops showing normalized muscle force ${\hat{F}}_{m}$ versus normalized muscle length ${\hat{l}}_{m}$ for each simulation. Simulations with *u*_max_ of 0.1 are shown in panels A and C, and simulations with *u*_max_ of 1 are shown in B and D. The non-dimensional muscle forces and lengths are identical for simulations with $\lambda_{l}{}_{{}_{0}}$ of 1 (A,B) and 10 (C,D).

All simulations resulted in muscle length, velocity, force, and power outputs that qualitatively resemble the behaviour of *in vivo* muscle during cyclic contractions where the muscle is generating mechanical power (Figs 3 and 4). Faster muscles with ${\dot{\varepsilon }}_{0}$ of 10 s^-1^ generated greater average mass-specific power per cycle P^*^ than slower muscles with ${\dot{\varepsilon }}_{0}$ of 5 s^-1^ at a given cycle frequency *f*. A higher ${\dot{\varepsilon }}_{0}$ allows muscle to generate more force at a given contraction velocity, which translates to greater power. Additionally, faster muscles generate greater P^*^ because they have faster rates of activation and deactivation than slower muscles. Theoretically, a muscle would generate the greatest P^*^ if it could activate and deactivate instantaneously at the beginning and end of the shortening phase of the contraction cycle. However, *in vivo* activation and deactivation is not instantaneous, and therefore muscle is activated before reaching peak length and deactivated while shortening to maximize mechanical work and P^*^ [50], consistent with the behaviour of our model (Fig 3). These delays to peak activation and relaxation contribute to reduced P^*^ for simulations with higher values of *f*, particularly for slower muscles with greater τ_act_ where there is insufficient time in the shortening phase for the muscle to reach full activation, and insufficient time to fully deactivate during lengthening.

Some unexpected effects also occurred as a result of assumptions made in developing the system. The maximum excitation *u*_max_ scales the forces in the muscle and therefore the power, so higher *u*_max_ resulted in higher P^*^ values. However, this effect was greater than that predicted from sinusoidal contraction cycles about optimal length [51] due to a shift in the operating range of muscle lengths at different values of *u*_max_. The muscle model contracted primarily on the ascending limb and plateau of the active force-length relationship when *u*_max_ was 1, and on the descending limb when *u*_max_ was 0.1. This effect is likely a consequence of the forward dynamics nature of the simulations where the muscle lengths respond to the dynamics of the contraction. Lower mean ${\hat{F}}_{m}$ at lower *u*_max_ results in the muscle being in a less contracted state, and thus operating at longer muscle lengths. This differs from *in situ* studies [51–57] where muscle is typically tested with contraction cycles centred about *l*_0_.

Hill-type muscle models are widely used within the field of biomechanics to predict muscle function in living animals where measurement is oftentimes not feasible. To evaluate the effects of different model formulations, Hill-type models are typically assessed within inverse dynamics frameworks using steady, non-cyclic kinematics. However, such simulations are limited in their ability to assess how changing different muscle properties impacts the behaviour of muscle, including work and power output during cyclic contractions. The framework in this study will provide a testing platform whereby current and future formulations of Hill-type muscle models can be tested under common contractile regimes that emulate the contractions cycles typical in locomotion. This framework is also consistent across scales, and so can be used to reconcile information from single fibre to whole muscle experiments. Future work could utilize this methodology to evaluate the relative influence of effects such as history-dependent, internal mass, activation, and tendon effects on the behaviour of muscle during cyclic contractions under a wider range of cycle frequencies, excitations, and loading conditions.

## Supporting information

S1 Table Optimized parameter values. (XLSX)

S2 Table Force-velocity Bézier control points. (XLSX)

S3 Table Active force-length Bézier control points. (XLSX)

S4 Table Passive force-length Bézier control points. (XLSX)

S1 Dataset Raw simulation output data. (ZIP)
